# Study of the Neuroprotective Effects of Memantine in Patients with Mild to Moderate Ischemic Stroke 

**Published:** 2014

**Authors:** Hamidreza Kafi, Jamshid Salamzadeh, Nahid Beladimoghadam, Mohammad Sistanizad, Mehran Kouchek

**Affiliations:** a *Department of Clinical Pharmacy, Faculty of Pharmacy, Shahid Beheshti University of Medical Sciences, Tehran, Iran. *; b *Department of Neurology, Imam Hossein Medical and Educational Center, Shahid Beheshti University of Medical Sciences, Tehran, Iran. *; c *Imam Hossein Medical and Educational Center, Shahid Beheshti University of Medical Sciences, Tehran, Iran.*

**Keywords:** Memantine, Ischemic stroke, NIHSS, Neuroprotection

## Abstract

Ischemic stroke is amongst the top four causes of mortality and the leading cause of disability in the world. The aim of this study was to evaluate the efficacy of a high dose memantine on neurological function of patients with ischemic stroke.

In a randomized, 2 armed, open-label study, patients with mild to moderate cerebral thromboembolic event (CTEE) who admitted to Imam Hossein Hospital, Tehran, Iran, during preceding 24 hours, entered the study. Patients allocated in two study groups of memantine (as add-on therapy) and control. All patients were managed based on the American Heart Association and American Stroke Association (AHA/ASA) guidelines. Patients in memantine group received conventional treatment plus memantine 20 mg TID. The National Institute of Health Stroke Scale (NIHSS) was determined and recorded daily. The primary objective was comparison of the changes in NIHSS in the study groups at day 1 and day 5 of intervention. Significance level of p<0.05 was considered for statistical analysis.

Patients were randomly allocated in control (15 women and 14 men, age 70.78 ± 10.92 years) and memantine (16 women and 8 men, age 73.33 ± 9.35 years) groups. There were no significant differences in age and sex distribution of two study groups as well as in comorbidities and concurrent drugs. NIHSS changes were significantly different between control (1.24 ± 0.96) and memantine group (2.96 ± 0.1), (p < 0.0001).

Our results reveal that memantine added to standard treatment of CTEE could result in a remarkable decrease in the NIHSS confirming improvement of the neurological function of the patients.

## Introduction

When considered separately from other CVDs, stroke ranks number 4 among all causes of death, behind diseases of the heart, cancer, and CLRD (chronic lower respiratory disease). Each year, in the USA, about 795 000 people experience a new or recurrent stroke. Approximately 610 000 of these are first attacks, and 185 000 are recurrent attacks. On average, every 40 seconds, someone in the United States has a stroke, and every 4 minutes, someone dies of a stroke (1).

The economic impact of this disease is enormous. The American Heart Association/American Stroke Association estimated that between 2012 and 2030, total direct annual stroke-related medical costs are expected to increase from $71.55 billion to $183.13 billion. Real indirect annual costs (attributable to lost productivity) are projected to rise from $33.65 billion to $56.54 billion over the same period. Overall, total annual costs of stroke are projected to increase to $240.67 billion by 2030. In this project, costs associated with other cardiovascular diseases (hypertension, coronary heart disease, and congestive heart failure) were excluded (2).

With an ageing population, established treatments for ischemic stroke are required to limit the extent of stroke-induced morbidity and mortality. Unfortunately, efficacy of only one pharmacological agent, *i.e. *recombinant tissue plasminogen activator (rtPA), has been approved for use in patients suffering acute ischemic stroke.rtPAis a thrombolytic agent that provides recirculation of the blood flow to the ischemic brain (3-5). Reports shows that only a low proportion of patients (around 2%–5% of cases) receive rtPA treatment due to restricted eligibility criteria such as time that passes after a stroke (3, 6). Although, it is possible to restore the blood flow to the ischemic brain by thrombolysis and subsequent delivery of oxygen and nutrients, however, because of limited means of reduction in neuronal death, targeting the brain parenchyma with pharmacological compounds may also be a promising strategy to control the spread of infarcted tissue(7).

Neuroprotection is specifically defined as the “protection of neurons” and is a strategy used topotentially protect the brain in a number of different cerebral conditions including Parkinson’s disease, traumatic brain injury and ischemic stroke(-). A neuroprotectant is mainly defined as an agent that prevents neuronal death by inhibiting one or more of the pathophysiological steps in the processes following injury to the nervous system or ischemia due to occlusion of an artery or hypoxia due to any cause (11).

Following cerebral ischemia, a complicated cascade of biochemical events occurs, eventually leading to the loss of neurons. Within this cascade, many molecular targets can be pharmacologically modulated to createneuro protection (12). Some of the molecular events that can be aimed by neuroprotectants include: glutamate release, glutamate receptor activation, excitotoxicity, Ca^2+^ influx into cells, mitochondrial dysfunction, activation of many intracellular enzymes, free radical production, nitric oxide production, apoptosis, and inflammation (10). Over 1000 potential neuroprotective therapies have been studied, in preclinical trials targeting some of these molecular events, with many of them providing protection (13).

There are also a number of ongoing trials for neuroprotective strategies including hypothermia and albumin, however the outcome of these approaches remains to be seen (10). In recent years, neuroprotective effects of some herbal medicines have been considered in several studies (14, 15)

As restoration of oxygen and glucose will always be the best therapy to protect against cell death from stroke, combination therapies with thrombolysis are also investigated in different studies.

There are also several promising neuroprotectants such ashematopoietic growth factors, and inhibitors of the nicotinamide adenine dinucleotide phosphate oxidases in preclinical investigation (10).

Memantine has been approved for the treatment of Alzheimer disease (AD) and has a good safety record. The molecular basis for memantine efficacy in neurological disorders is at least in part due to the improvement of over-activation of NMDA *(N-methyl-D-aspartate)*receptors that causes excessive Ca^2+^ influx through the receptor’s associated ion channel and subsequent free radical formation. 

In experimental studies in rats, it was shown that memantine infusion at low doses can lead to steady-state serumlevels within a therapeutic range which can provide neuroprotection as well as cognitiveenhancement (11). In an *in-vitro* study, Montagne *et al*. showed that memantine is able to prevent the pro-neurotoxic effects of rtPA in cultured cortical neurons. This study revealed that memantine blunted the noxious effects of delayed thrombolysis on lesion volume and neurological deficit after ischemic stroke. In addition, memantine rescued rtPA-induced decrease in survival rate after intracerebral hemorrhage (16).

Though, neuroprotective effects of memantine in stroke has been confirmed by different animal studies, nevertheless, to the best of our knowledge, there are no studies regarding its efficacy in stroke patients. 

The present study was performed to assess the effects of memantine on neurological function of patients suffering from ischemic stroke. Neurological function was assessed by the NIHSS (National Institute of Health Stroke Scale) score. This scale could be used as a clinical stroke assessment tool to evaluate and document neurological status in acute stroke patients. The NIHSS is valid for predicting patient’s outcome and can serve as a measure of stroke severity (17).The NIHSS has been shown to be a predictor of both short and long term outcome of stroke patients. Additionally, this stroke scale may serve as a data collection tool for planning patient care and could provide a common language for information exchanges among healthcare providers (3, 18). NIHSS classifies stroke as mild (scores less than 8), moderate (scores between 8 to16) and sever (scores higher than 16). 

## Experimental

*Study design and setting*

In a prospective, randomized, open label, 2-arm parallel-groupstudy, adult patients with ischemic stroke was recruited in two groups: control group and memantine group. The study was carried out between September 2011 and December 2012 in the neurology ward of Imam Hussein hospital, affiliated to the Shahid Beheshti University of Medical Sciences, Tehran, Iran. Patients in control group treated based on standard treatment recommended by the AHA and ASA (American Heart Association and American Stroke Association) guidelines (19). All patients with ischemic stroke should receive statin. Ambolic strokes should be managed by warfarin. Aspirin, clopidogrel, ticlopidine and dipyridamole are drugs which administered to prevent recurrence of ischemia. Heparin or Enoxaparin could be used to deep vein thrombosis in susceptible patients. Family and patient education about stroke and daily physiotherapy services were done for patients.

Patients in memantine group received memantine 20 mg three times per day (two 10 mg tablets, batch number 0021110, Exir^® ^Pharmaceuticals, Iran) for five days in addition to the standard treatment. Patient’s data including NIHSS score recorded at entrance to study and at day five of the study.

The study protocol was registered, reviewed and approved by Iranian Registry of Clinical Trials (IRCT), with registry number of IRCT2012092910178N2. IRCT is listed as a primary registry at the WHO International Clinical Trials Registry Platform. 

*Patients*

The population in the present study was comprised of patients with a computerized tomography (*CT*) *scan*proven ischemic stroke. All patients were given a written informed consent before entering the study, and the study was performed in accordance with the declaration of Helsinki. The ethics committee of Shahid Beheshti University of Medical Sciences approved the study.

*Inclusion criteria*

- all patients (male or female) with mild to moderate ischemic stroke based on NIHSS score (0 to 16),
- hospital admission within 24 hours of onset of stroke symptoms,
- no age limit was considered.

*Exclusion criteria*

- included hemorrhagic stroke (based on CT scan findings),
- hyper reactivity to memantine or any ingredients in the tablet formulation,
- acute or chronic renal failure stage 4 and 5 (based on Acute Kidney Injury Network (AKIN) criteria),
- moderate to severe hepatic disease (based on Child-Pugh criteria, grade B and C),
- acute MI (<48 hours),
- autoimmune diseases,
- Alzheimer disease or any kind of dementia,
- history of memantine intake during 6 months before stroke.

*Data analysis*

The Statistical Package for the Social Sciences (SPSS, version 19.0; IBM company, USA) was used for data analysis. Statistical tests of the chi-square, the Fisher’s exact test, the Mann-Whitney u-test and student t-test were applied to analyze the data obtained from study groups. p<0.05 was considered as the significance level.

## Results

**Figure 1:**
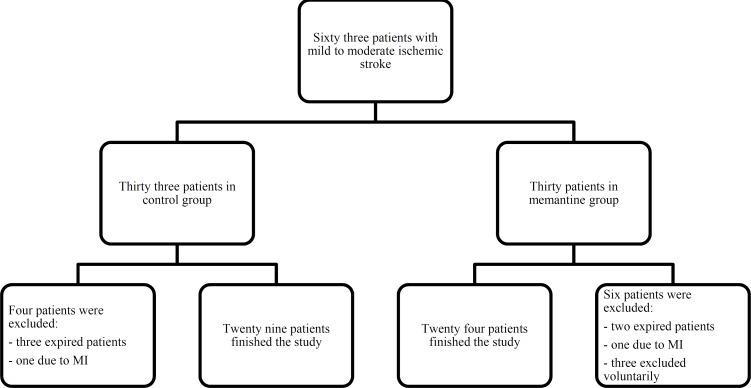
Flow chart of the study participants

Sixty three patients with mild to moderate ischemic stroke and NIHSS 0 to16, were enrolled in the study. Three patients were excluded voluntarily, five patients expired during the first five days of hospital stay and two patients excluded because of acute MI. The study completed with twenty nine patients in the control group and twenty four patients in the memantine group. The clinical trial flowchart process is presented in Figure 1.

The control group included 15 (51.7%) women and 14 (49.3%) men with mean ± SDage of 70.78 ± 10.92 years and the memantine group consisted of 16 (66.7%) women, 8 (33.4%) men with mean ± SD age of 73.33 ± 9.35 years. No significant differences were observed between sex (p = 0.27) and age (p = 0.43) distribution of two study groups. Most common comorbidities in patients were diabetes mellitus, atrial fibrillation, angina pectoris, hypertension, hyperlipidemia and migraine headache, with no significant differences (p = 0.73) between two groups. Also, no significant difference was observed between medications received by patients in two study groups (p = 0.97). Most common administered drugs were: captopril, enalapril, losartan, valsartan, aspirin, atorvastatin, atenolol, metoprolol and clopidogrel. Table 1 represents the medical history of the study groups and their medication prescribed during hospitalization.

Within group analysis showed that in the control group, differences between baseline NIHSS score (10.14 ± 3.46) and that of the day 5 (8.90 ± 3.53) was significant (p<0.0001; 95% CI: 0.88-1.60). Similarly, in the memantine group there was a significant difference (p<0.0001; 95% CI: 2.54-3.38) between baseline NIHSS score (10.25 ± 3.12) and that of the day 5(7.29 ± 2.65). However, between groups comparison revealed that NIHSS score change from day 1 to day 5 was significantly different between control (1.24 ± 0.96) and memantine group (2.96 ± 0.1), (p < 0.0001).So that patients in the memantine group have significantly greater reduction in the NIHSS score compared to that of the control group.

The most common adverse reaction of memantine was nausea reported in 25% of patients which were the reason of non-adherence to high dose memantine resulting in drop-out of three patients.

**Table 1:** Demographic, NIHSS change, comorbidities and drug history of patients in control and memantine groups.

	Memantine	Control	p-value
Sex			
**Men**	8	14	0.27
**Women**	16	15	
**Age, median (interquartile range)**	75 (64.25-81.75)	71(67-80)	0.43
**NIHSS change from day 1 to day 5**	2.95±0.99	1.24±0.95	<0.0001
**Medical history** ***DM***	11	14	0.73
***HTN***	15	12	
***HLP***	19	13	
***AF***	9	7	
***CHD***	7	11	
***Migraine ***	3	3	
**Medication** ***ACEI***	3	3	0.97
***ARB***	7	5	
***Clopidogrel***	4	3	
***ASA***	15	17	
***Warfarin***	7	9	
***Dipyridamole***	3	3	
***Atorvastatin ***	24	29	
***Beta blocker***	11	11	
***Diuretics ***	2	2	
***Anticoagulant ***	12	15	
***Metformin ***	7	11	
***Sulfonyl urea***	9	9	
***Insulin ***	2	5	
***Amlodipin***	4	1	

DM: Diabetes Mellitus; HLP: hyperlipidemia; AF: atrial fibrillation; CHD: coronary heart disease; HTN: Hypertension; ACEI: angiotensin convertase enzyme inhibitor; ARB: angiotensin receptor blocker

## Discussion

Although, there is no controlled study using memantine in acute phase of stroke in human, many animal studies confirmed its neuroprotective pharmacologic effects in higher doses. Therefore, a prospective, randomized, open label, 2-arm parallel-group study was designed to investigate the use of approved higher doses of memantine in patients at acute phase of stroke. To evaluate the effectiveness of memantine on minimization of the neurologic sequels in post-stroke patients, the NIHSS was considered as the primary outcome measure. Our results showed that 60 mg daily doses of memantine (20 mg TID) could be beneficial for neurologic function improvement of patients with ischemic stroke, as confirmed by NIHSS changes in the study groups.

Memantine has been investigated extensively in animal studies and its safety has been established and confirmed by clinical experience in human (11).

Dogan *et al*. showed that memantine is effective in preventing neuronal damage after permanent focal cerebral ischemia. In this study Spontaneously Hypertensive Rats (SHR) weighing 250-300 g were anesthetized with halothane and subjected to 1 hour of temporary middle cerebral artery occlusion by an intraluminal suture. 20 mg/Kg of memantine or saline were injected intraperitoneally 5 minute after the induction of ischemia. Treatment with 20 mg/Kg memantine (n=14) reduced the ischemic injury volume to 233+/-61 mm (3) (P<0.01). The results demonstrate that the harmful effects of recirculation after a period of ischemia can be attenuated by the treatment of memantine, perhaps by its action at the NMDA receptors (20)

In a study conducted by Volbracht *et al.*, neuroprotective properties of memantine were approved in different *in-vitro* and *in-vivo* models of excitotoxicity. As expected, memantine protected neurons in organotypic hippocampal slices or dissociated cultures from direct NMDA-induced excitotoxicity. However, low concentrations of memantine were also effective in neuronal (cortical neurons and cerebellar granule cells) stress models dependent on endogenous glutamate stimulation and mitochondrial stress, *i.e*. exposure to hypoxia, the mitochondrial toxin 1-methyl-4-phenylpyridinium (MPP+) or a nitric oxide (NO) donor. Furthermore, memantine reduced lethality and brain damage *in-vivo* in a model of neonatal hypoxia-ischemia (21).

Lapchak *et al*. showed that memantine 10 mg/Kg infusion improves clinical rating scores in a multiple infarct embolic stroke model in rabbits. The investigators used a rabbit multiple infarct ischemia model with a well-defined behavioral endpoint. In this study, results suggest that uncompetitive NMDA antagonists, more specifically open channel blockers, which may be alternatives to high affinity NMDA antagonists, may have substantial therapeutic benefit for the treatment of acute ischemic stroke. They suggested that memantine or new dual activity analogs of memantine should be further pursued as a useful therapy to treat the behavioral deficits associated with multiple-infarct ischemia (22).

Results of our study in accordance with findings of animal studies showed that memantine could improve neurological function – measured by NIHSS- in patients with ischemic stroke.

The approved dosing of memantine in Alzheimer disease is step by step increasing the dose to maximum 20 mg daily (11). In animal studies dosage of memantine which showed neuroprotective properties was 5-50 mg/Kg (23-26). The human equivalent dose based on these studies for a 70 Kg person is 56.7-567 mg/70Kg daily by translation of the animal dosage based on body surface area.In human studies (for disease other than stroke), maximum administered dose of memantine was 60 mg daily (27-29). Since, higher dosage of neuroprotectant is crucial at the first days of stroke, at present study memantine 60 mg daily was administered for the first five days after stroke. It should be contemplated that the average hospitalization time in the neurology ward of the Imam Hossein hospital, as the study setting, is five days. We did not administer high dose memantine after discharge from hospital because patients could not be observed and followed for possible adverse reactions due to memantine.

Our results are in accordance with a study by Berthier *et al*. who studied memantine in post stroke aphasia and reported its effectiveness. This randomized, double-blind, placebo-controlled, parallel-group study of both memantine and constraint-induced aphasia therapy (CIAT) on chronic post-stroke aphasia followed by an open-label extension phase, showed both memantine, 20 mg/day, and CIAT alone improved aphasia severity, however the best outcomes were achieved with combining memantine with CIAT. Beneficial effects of memantine and CIAT persisted on long-term follow-up (30).

Memantine at a clinically relevant dose also markedly increased BDNF (Brain Derived Neurotrophic Factor) mRNA levels in the limbic cortex. This effect was more widespread and pronounced at higher doses. Thus the neuroprotective properties of memantine could be mediated by the increased endogenous production of BDNF in the brain (11). 

Although,in studies with doses of memantine higher than 20 mg/day no significant side effects have been reported, 25% of patients in our study experienced nausea which was, of course, endurable in majority of the patients.

Collines *et al.* investigated the acute effects of pretreatment with high-dose memantine, on the effects of cocaine in humans. Six African American men completed this laboratory study, in which, following pretreatment with memantine (0 or 60 mg), no significant side effect due to memantine was reported (27).In a study performed by Bisaga *et al*. participants were randomized to receive either memantine 20 mg bid (N=39) or placebo (N=42) for 12-weeks in combination with individual relapse-prevention therapy. The efficacy of memantine 40 mg/day for the treatment of cocaine dependence was not supported. Nausea was reported only in 5.1% of subjects vs. 4.8 of placebo (28). 

In another study by Bisaga *et al*., eight heroin-dependent, non-treatment seeking, in-patient participants were stabilized on a fixed dose of morphine (30 mg PO qid). They also received a series of challenges with naloxone (0.4 mg, IM) and after ward the severity of opioid withdrawal was monitored. Either placebo or memantine (60 mg PO) was given 6 h before each naloxone challenge. A modified multiple base line, across-participants design was used to evaluate the effects of memantine on the severity of naloxone-precipitated opioid withdrawal. Memantine attenuated the expression of opioid physical dependence in humans, indicating that glutamatergic neurotransmission at the NMDA receptor site contributes to the maintenance of opioid dependence(28). There was no report of significant adverse reaction caused by memantine, in this study.

Memantine can cause nausea and vomitingat regular dosages in 5.1% and 7.1% of patients, respectively. Higher rate of nausea in our study could be due to advanced age, poly pharmacy due to comorbidities, using multiple doses of memantine (20 mg, TID) and their difficult medical condition of stroke.

## Conclusion

Incompliance with the previous researches performed to study of the neuroprotective effects of memantine and its mode of action in different cerebral disorders, our findings confirm that a-five day post-stroke treatment with memantine 20 mg TID could have significant valuable effects on the neurological consequences of stroke and the neurologic function improvement of the study patients. However, studies with a larger sample size and longer fallow up periods are recommended in order to more evident demonstration of the neuroprotective effects of memantine in ischemic stroke. Also, studies to assess neurological biomarkers and different doses of memantine are other recommendations that need to be considered in future studies.
